# Electrochemical Evaluation of Protective Coatings with Ti Additions on Mild Steel Substrate with Potential Application for PEM Fuel Cells

**DOI:** 10.3390/ma15155364

**Published:** 2022-08-04

**Authors:** Diana N. Avram, Corneliu M. Davidescu, Mircea L. Dan, Julia C. Mirza-Rosca, Iosif Hulka, Alexandru Pascu, Elena M. Stanciu

**Affiliations:** 1 Faculty of Industrial Chemistry and Environmental Engineering, CAICAM Department, Politehnica University Timişoara, Blvd. V. Pârvan, nr. 6, 300223 Timişoara, Romania; 2 Renewable Energy Research Institute-ICER, Politehnica University Timisoara, 138 Gavril Musicescu Street, 300774 Timişoara, Romania; 3 Department of Mechanical Engineering, Las Palmas de Gran Canaria University, 35017 Las Palmas de Gran Canaria, Spain; 4 Materials Engineering and Welding Department, Transilvania University of Brasov, Eroilor Blvd., 29, 500036 Brasov, Romania

**Keywords:** protective coatings, Ti addition, laser cladding, corrosion, PEMFC

## Abstract

In this work, the corrosion behavior of NiCr(Ti) protective coatings deposited on mild steel substrates through laser cladding technology is studied as an alternative new material for metallic bipolar plates used in PEMFC applications. For electrochemical testing, a solution consisting of 0.5 M H_2_SO_4_ + 2 ppm F^−^ at room temperature is used as an electrolyte. The fluoride ions are added to simulate the conditions in the PEM fuel cell due to degradation of the proton exchange membrane and fluoride release. A saturated calomel electrode (SCE) is used as a reference electrode and a platinum mesh as the counter electrode. Scanning electron microscopy (SEM) and optical microscopy (OM) are used for studying the morphology of the protective coatings and the effect of Ti addition. The electrochemical evaluation consisted of measuring the open circuit potential (OCP), followed by electrochemical impedance spectroscopy measurements (EIS) and potentiodynamic polarization. It is found that the coatings with 5% Ti, 7% Ti and 10% Ti addition comply with the conditions of the US DOE regarding corrosion performance to be used as materials for the manufacture of the bipolar plates.

## 1. Introduction

Every year we see an increasing energy demand in our society with a negative impact on the environment and the generation of greenhouse gas emissions (essentially CO_2_). The need for clean methods of electricity production has stimulated the search for new sources of sustainable energy. Fuel cells (FCs) represent an attractive solution because of their ability to convert chemical energy directly into electrical energy for as long as fuel and oxidants are supplied [1]. In the transportation sector, internal combustion engines (ICEs) have come to have a competitor in fuel cells due to the fact that FCs are electrochemical engines with zero or very low emissions. Thus, they have been named zero-emission engines [2]. Among different types of fuel cells, polymer electrolyte membrane fuel cells (PEMFCs) have acquired a lot of attention for transport applications due to their high efficiency, relatively low temperature, high power density and eco-friendly performance [3,4]. The bipolar plates (BPs) are one of the most important components of a PEMFCs that comprise approximately up to 80% of the total volume and up to 45% of the cost of a fuel cell stack. The BPs have important functions such as uniform distribution of fuel and oxidant gases within the cell, collecting electrical current and transporting it through the fuel cell stack, separating the individual cells in the stack and removing exhaust water and heat out of the stack [5]. According to the US Department of Energy (US DOE), the materials from which the bipolar plates are manufactured must satisfy minimum specifications, such as the following: high corrosion resistance, high electrical and thermal conductivity, low cost, high mechanical strength, easy manufacturing, low gas permeability and low resistivity [6]. The US DOE targets bipolar plates for transportation applications in 2020 regarding the corrosion behavior. It stipulates that the corrosion rate on both the anodic and cathodic sides must not exceed 1 µA/cm^2^ and that the anodic side must not have active peaks [6]. In the early stages of PEMFC development, impregnated graphite was used as a bipolar plate material due to its low contact resistance and good corrosion resistance. However, because of its poor mechanical properties, high porosity and gas permeability, research has shifted to metallic bipolar plates (MBPs). Compared to graphite, metallic bipolar plates satisfy the US DOE requirements in terms of manufacturing and mechanical proprieties but are more susceptible to surface corrosion and dissolution of metallic ions in the PEMFC environment [7].

Protective coatings have been investigated in order to improve the corrosion resistance of metallic bipolar plate surfaces. In recent years, different manufacturing methods of protective coatings have been studied, such as the following: chemical vapor deposition [8], magnetron sputtering [9], cathodic arc deposition technique [10], electron beam evaporation [11], arc ion plating [12], high-velocity oxygen fuel (HVOF) [13,14], pulsed laser deposition [15,16], etc. Among the deposition techniques, laser cladding (LC) is a strong candidate for the manufacturing of coatings with enhanced surface proprieties. It has the advantage of applying a different material as a coating onto the surface of the selected substrate [17,18]. Recent studies have shown that laser cladding can be an efficient tool to produce high-density coatings on large surfaces with small heat-affected zones and improved corrosion and wear resistance. The deposition of a wide range of materials has been successfully performed using various materials for different applications. Considerable attention is attributed to NiCr-based superalloys due to their ability to provide high-quality coatings, anti-corrosive properties and their cost-effectiveness [19,20,21]. MetcoClad 625F is a non-magnetic nickel-chromium-molybdenum-based superalloy, similar to Inconel 625. The presence of nickel and chromium in the alloy provides good corrosion resistance in oxidizing environments, while the presence of molybdenum provides good corrosion resistance in non-oxidizing environments. The formation of Cr2O3 film through passivation on the alloy surface has been reported to be the primary reason for the corrosion resistance of the MetcoClad 625F superalloy. Moreover, the presence of 8–10 wt.% Mo in the alloy enhances the resistance to pitting and crevice corrosion [22,23]. For further improvement, the introduction of reinforcing particles into the metal matrix increases the mechanical performance and deposition quality. In some publications [24,25,26], titanium (Ti) has been added to the commercially available raw materials and protective coatings have been successfully manufactured. It was observed that new phases were formed that improved the properties of the coatings in terms of corrosion resistance. Jin et al. [27] have studied CrTiN coatings with different Ti content deposited on stainless steel substrates by using magnetron sputter ion planting as bipolar plates for proton exchange membrane fuel cells. The study showed that the Ti addition provided increased corrosion resistance.

In the present study, mild steel plates were coated with NiCr(Ti) protective coatings using laser cladding technology, in order to obtain new materials with potential application for bipolar plates in PEM fuel cells. The newly developed materials are expected to have increased corrosion resistance with the addition of Ti to the metal matrix. Thus, the effect of Ti addition on the microstructure of the coatings was studied by using optical microscopy (OM) and scanning electron microscopy (SEM). The phases developed within the coatings were studied by X-ray diffraction (XRD) and the electrochemical behavior was studied using open circuit potential (OCP), electrochemical impedance spectroscopy (EIS) and potentiodynamic polarization techniques. To the best of our knowledge, laser-cladding NiCr-based alloys with Ti addition have not been studied in potential applications for bipolar plates for PEM fuel cells.

## 2. Materials and Methods

### 2.1. Feedstock Powder and Powder Deposition

The powder used in this study was obtained by mixing the commercially available MetcoClad 625F powder (particle size distribution −53…+20 µm) with different wt.% Metco 4010A powder (Ti, 99% purity with particle size distribution in the range of −90…+22 µm), both of them supplied by Oerlikon Metco, Switzerland. The MetcoClad 625F powder has the following composition according to the manufacturer: 62–69% Ni, 20–23% Cr, 8–10% Mo, 3–5% Nb and traces of iron and other elements. For a homogenous mixture, the powders were mechanically blended for 5 min at a speed of 150 rpm. Low carbon mild steel plates (60 mm × 25 mm × 5 mm) were used as a substrate for the deposition of feedstock powders. The base MetcoClad 625F coating (labeled B from now on) was reinforced by adding 1.5, 3, 5, 7 and 10 wt.% of Ti to the composition.

**Figure 1:**
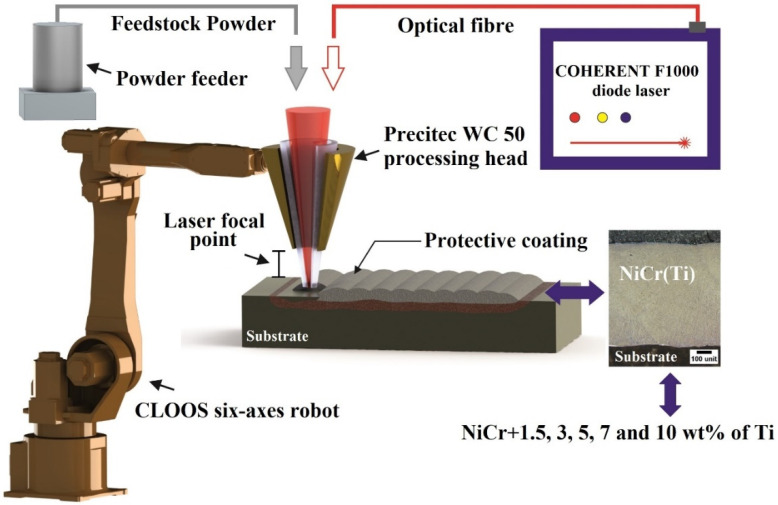
Schematic representation of the laser cladding process.

The experimental part was carried out using a COHERENT F1000 (Santa Clara, CA, USA) diode laser (λ = 975 nm) equipped with a PRECITEC WC 50 (Gaggenau, Germany) processing head (200 mm focal length). The cladding head was manipulated using a CLOOS six-axes robot and an AT-1200 HPHV TERMACH (Haiger, Germany) feeding system was used to transport the powder to the cladding head. Argon with 99% purity was employed as shielding gas. The schematic representation of the laser cladding process is presented in Figure 1.

Prior to laser cladding deposition, the mild steel substrates were polished to remove the oxides from their surfaces. On the prepared mild steel substrate, ten partially overlapped tracks with an overlap degree of 45% were deposited using the process parameters presented in Table 1.

### 2.2. Characterization of NiCr(Ti) Protective Coatings

The morphologies and microstructures of the laser-cladded coatings were analyzed in cross-section by optical microscopy (Axio Vert.A1, Carl Zeiss, Germany) and scanning electron microscopy (SEM, Quanta FEG 250, FEI, Hillsboro, OR, USA). Energy Dispersive X-ray Spectrometer (EDS, EDAX Inc., Mahwah, NJ, USA) was used for elemental analysis determination. Phase identification and quantification were determined using an X-ray Diffractometer (PANalytical X’Pert Pro powder, Malvern Panalytical, Malvern, UK) with CuKα radiation.

Corrosion resistance of the laser-cladded coatings was studied in a conventional three-electrode configuration electrochemical cell connected to a BioLogic potentiostat/galvanostat model SP-150 (BioLogic Science Instruments, Seyssinet-Pariset, France). An SCE electrode was used as a reference electrode and platinum gauze was used as a counter electrode. The working electrodes consisted of mild steel and laser-cladded samples with an exposed surface area of 0.785 cm^2^. Before measurements, the surface of tested samples had been polished with different grades of SiC abrasive papers (up to 2400 grit size). Afterward, they were polished using 3 μm diamond suspension until a mirror-like surface was obtained. Finally, the samples were cleaned with distilled water and ethanol. The electrolyte used in all electrochemical tests was a solution of H_2_SO_4_ 0.5 M (pH = 0.3) + 2 ppm F^−^ at room temperature. The fluoride ions were added to simulate the conditions in PEM fuel cell. During the operation of PEM fuel cells fluoride ions are released due to the degradation of proton exchange membrane and sometimes these ions can be adsorbed on organic materials [28].

Electrochemical evaluation of NiCr(Ti) coatings consisted of measuring the open circuit potential (OCP) for 2 h. Afterward, electrochemical impedance spectroscopy (EIS) studies were performed using the impedance module of Biologic SP-150 as described in our previous papers [29,30]. The frequency range was 100 mHz–100 kHz with an alternative voltage amplitude of 10 mV. In each test, 60 experimental data were recorded and represented by a logarithmic arrangement of 10 data points per decade. The recorded EIS data were fitted with an equivalent electrical circuit (EEC) by a complex non-linear least squares Levenberg-Marquardt procedure using ZView 2 software (Scribner Associates, Inc., Southern Pines, NC, USA). Potentiodynamic polarization curves were recorded at a scan rate of 1 mV/s in the potential range of −250 mV to +250 mV versus the OCP value.

## 3. Results and Discussion

### 3.1. Microstructure and Phase Analysis

**Figure 2:**
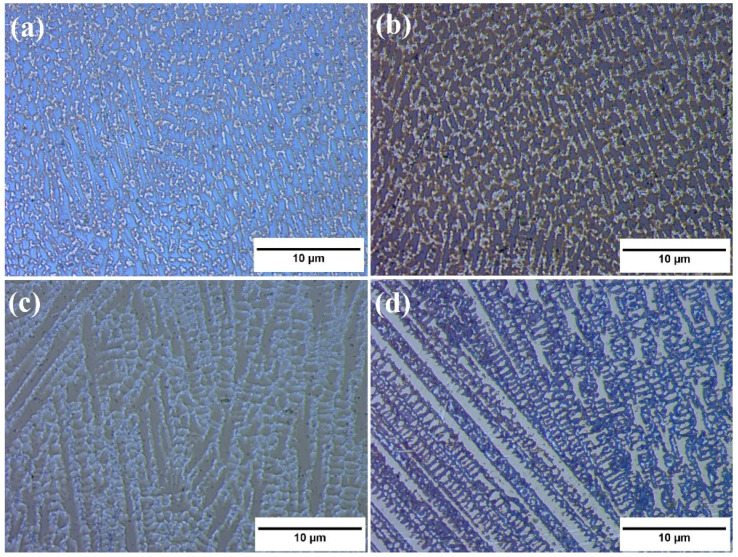
Microstructure of deposited coatings, electrochemical etched with oxalic acid, in cross section: (**a**) NiCr-coating without Ti addition; (**b**) with 3% Ti addition; (**c**) 5% Ti addition and (**d**) 10% Ti addition.

In order to visualize the microstructure of NiCr(Ti) coatings, the prepared samples were electrochemically etched with a 10 wt.% solution of oxalic acid at 3 V for 12 s. Representative metallographic images of coatings collected in cross-sections without Ti and with 3%, 5% and 10% Ti addition are presented in Figure 2. One can observe that the micrographs of the deposited laser cladded samples showed a dense structure free of cracks, porosity or other defects. Besides, the micrographs show a dendritic structure revealed by the etchant. Furthermore, it was observed that the size of dendrites increased with the Ti addition while the volume fraction of the NiCr-ɣ phase diminished.

**Figure 3:**
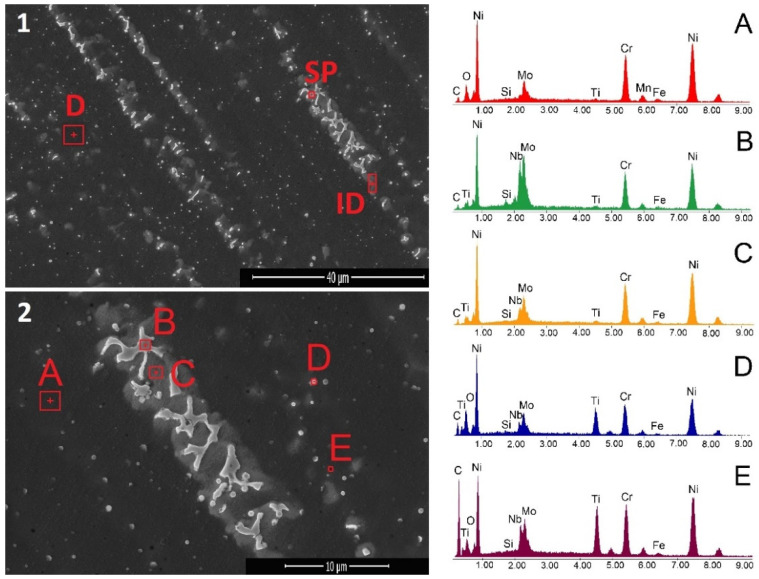
Representative SEM images of cross section NiCr laser-cladded coating with Ti addition at low (**1**) and high magnification (**2**) with EDS spectrum of different phases (**A**–**E**).

Representative SEM images taken in a cross-section of a NiCr-based coating with Ti addition (B + 5% Ti) are presented in Figure 3. The microstructural examination reveals dendritic structures (D) with inter-dendritic regions (ID) and secondary products (SP). A semi-quantitative analysis was performed to reveal the chemical composition of the NiCr(Ti) coating on the areas marked from A to E. According to the EDX analysis, it can be noticed that the dendritic region, labeled A, is rich in Ni, Cr and Mo. Traces of Fe and Ti were noticed as well. Besides, O can be noticed, which might be present due to powder particle oxidation during in-flight. The inter-dendritic area, labeled C, is rich in Ni, Cr, Mo and Nb, while trances of Ti and Fe were detected. During the cooling process, secondary phases form as well. The formation of secondary phases is caused by the segregation of the alloying elements that are added to the NiCr-based powder in smaller amounts. Among them, laves (labeled B) rich in Nb and Mo were observed and precipitates rich in Ti and Nb, which might be attributed to TiC (labeled D) and NbC, respectively (labeled E). The results are in accordance with other research carried out on laser-cladded NiCr-based coatings [22,31].

**Figure 4:**
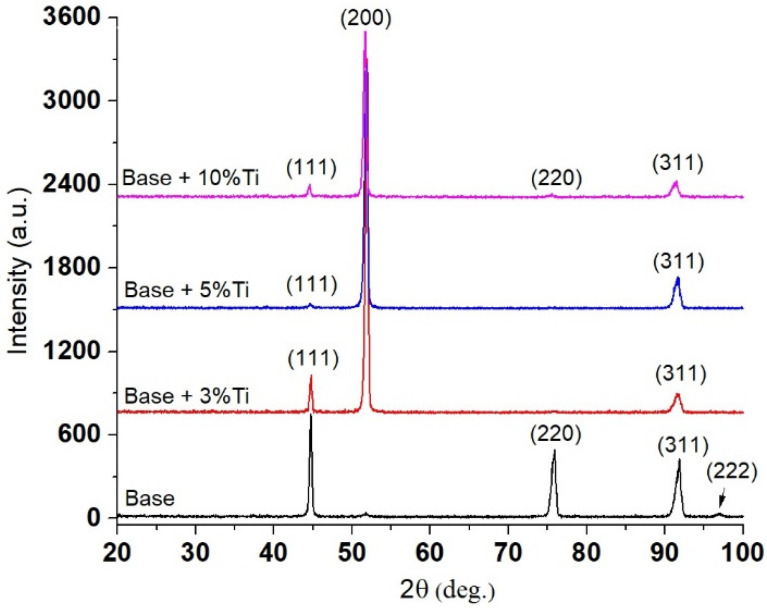
XRD spectrum of NiCr and NiCr(Ti) coatings with 3%, 5% and 10% Ti addition.

Phase identification was performed by XRD on NiCr-based coatings without Ti and with 3%, 5% and 10% Ti additions. In Figure 4 it can be seen that the peaks have different intensities due to the different orientations of grains caused by directional cooling. The XRD pattern of the NiCr-based coating was taken as a reference and is in accordance with data published in the literature [17,22]. The NiCr-ɣ phase diffraction peaks have been identified in all samples as follows: the first peak is (111) at 44.28°, the second one is (200) at 51.59°, (220) at 44.28°, (311) at 92.38° and (222) at 97.83° (PDF# 04-001-3422). In the coatings manufactured with Ti addition, the presence of Ti within the coatings was confirmed by the peak at the position of 51.59°. Titanium is dissolved into the FCC Ni-Cr phase and the new Ni-Cr-Ti phase can be attributed as well to the peak at the position of 51.59°. Other phases might have formed besides the ones identified, but due to their low intensity, they were not detected by XRD analysis.

### 3.2. Electrochemical Behavior

All metallic samples were electrochemically evaluated with the same protocol, which consisted of measuring for 2 h the open circuit potential (OCP) in the test solution, followed by electrochemical impedance spectroscopy measurements (EIS) and potentiodynamic polarization curves (Tafel method) at a low scan rate of 1 mV/s. All electrochemical determinations were performed at 25 °C.

**Figure 5:**
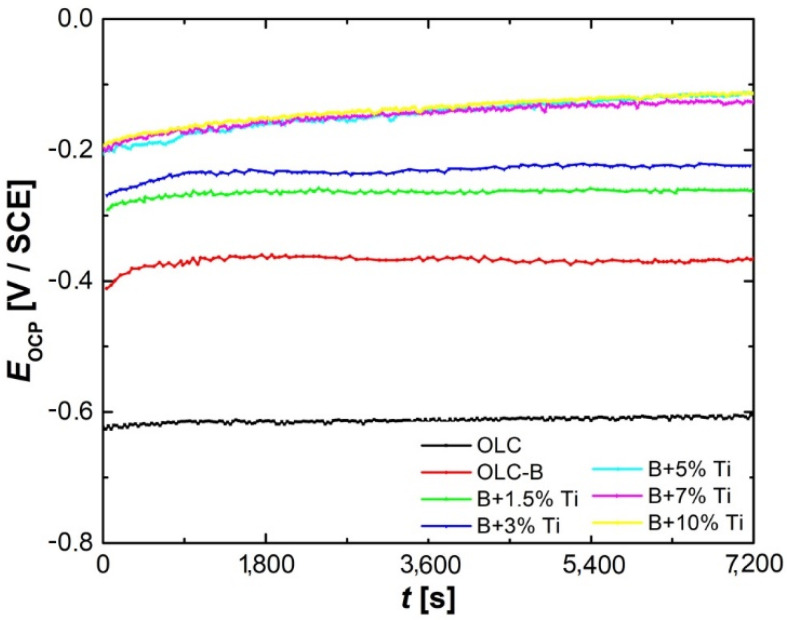
OCP evolution for mild steel without and with NiCr(Ti) coatings test samples recorded in test solution.

First of all, the OCP variation in time was measured in order to obtain preliminary information on the process that unfolds at the interface between the test electrode and acid electrolyte. The recorded OCP evolution in time for all tested samples is presented in Figure 5. The potential values after 2 h recorded in the absence of a current tend towards a cvasi-steady-state value, which is approximately equal to the corrosion potential values determined from the Tafel plots. For NiCr(Ti) coatings on mild steel, an OCP variation is observed towards more positive values with the increase in the titanium concentration added in the coating deposited on the OLC substrate. This variation of the OCP values is usually attributed to an increase in the corrosion resistance of the NiCr(Ti) deposited coatings. This is most likely caused by the formation of a passivating film on the surface of the laser-cladded coatings.

**Figure 6:**
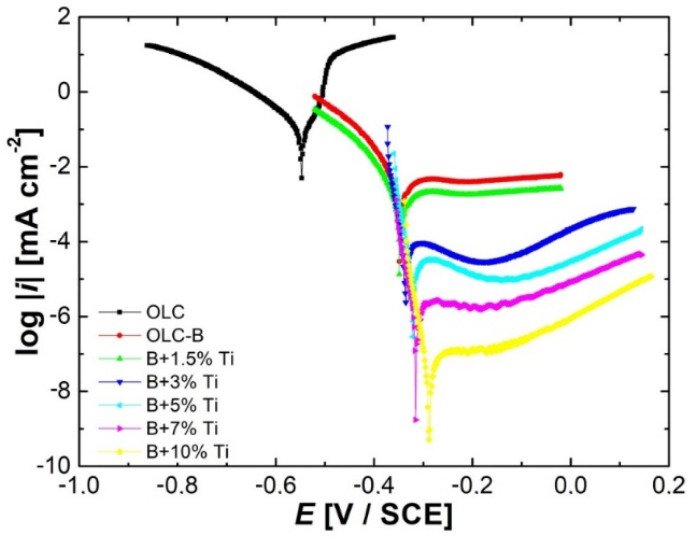
Tafel plots recorded at 1 mV/s scan rate for mild steel without and with NiCr(Ti) coatings test samples recorded in test solution.

The potentiodynamic polarization curves presented in Figure 6 obviously show a significant decrease in the anodic current densities with the increase in titanium addition in the NiCr(Ti) coatings, indicating an increase in corrosion resistance in the aggressive environment used in the experimental tests.

The same pronounced variation can be observed for the cathodic currents recorded for carbon steel used as a basis for the deposition of different layers and samples with applied NiCr(Ti) coating. The values of the cathodic current densities decrease proportionally with the amount of titanium added to the coatings. Therefore, this means that in the acidic environment used to carry out the tests, titanium acts as an inhibitor of the cathodic process, thus limiting the hydrogen evolution reaction.

Table 2 summarizes the values of polarization parameters such as the following: corrosion current (*i_corr_*), corrosion potential (*E_corr_*) and slops of the anodic (*b_a_*) and cathodic (*b_c_*), all these values being calculated using the Tafel extrapolation method with the BioLogic SP150 software, EC-lab 10.38. Moreover, the polarization resistance (*R_p_*) was calculated for all the investigated samples using the Stern–Geary Equation as follows (1):

(1) $$R_{p}=\frac{b_{a}\cdot b_{c}}{i_{corr}\cdot 2.303\left(b_{a}+b_{c}\right)}$$

where: *R_p_* is the polarization resistance, Ω·cm^2^; *b_a_* and *b_c_* are the anodic, respectively, the cathodic Tafel slopes, V; *i_corr_* is the corrosion current density measured in A·cm^−2^.

Analyzing the presented data, it can be seen that the best results were obtained for the coating with 5%, 7% and 10% Ti additions with a current density value below 1 µA·cm^−2^, which is the target set by the U.S. Department of Energy (U.S. DOE) in 2020 [6]. Even more, the corrosion current density values for the last two coatings are below 0.1 µA·cm^−2^. Besides, the coatings have the lowest *i_corr_* and highest *R_p_* values, indicating high anticorrosive proprieties.

The protective efficiency (*PE*) of the NiCr(Ti) coatings on mild steel in the test solution was calculated from the equation shown below as follows:

(2) $$PE\left(\%\right)=100\cdot (1-\frac{i_{corr}}{i_{corr}^{0}})$$

where $i_{corr}^{0}$ corresponds to the current density of the uncoated mild steel and *i_corr_* corresponds to the current densities of NiCr(Ti) coatings obtained from the potentiodynamic studies in the test solution. From Table 3, it can be seen that the protective efficiency increases with the increase in Ti content. The reason for this is the formation of a stable oxide film on the surface that enhances the corrosion resistance in an aggressive acidic medium. The corrosion rate (*CR*) of uncoated mild steel and NiCr(Ti) coatings was calculated with the following equation:

(3) $$CR=K_{1}\cdot \frac{i_{corr}}{\rho }\cdot EW\;\;$$

where *CR* represents the corrosion rate (mm per year), *i_corr_* the corrosion current density (µA·cm^−2^) obtained from the potentiodynamic studies. *EW* and *ρ* are the equivalent weight and densities of the samples (g·cm^−3^), respectively. *K*_1_ is a constant with a value of 3.27 × 10^−3^ (mm·g·µA^−1^·cm^−1^·yr^−1^). From Table 3, it can be seen that the corrosion rates decrease with the increase in Ti content due to the formation of the thin oxide film developed on the coating’s surface. It is well known that Ti spontaneously forms oxides when exposed to the environment.

**Figure 7:**
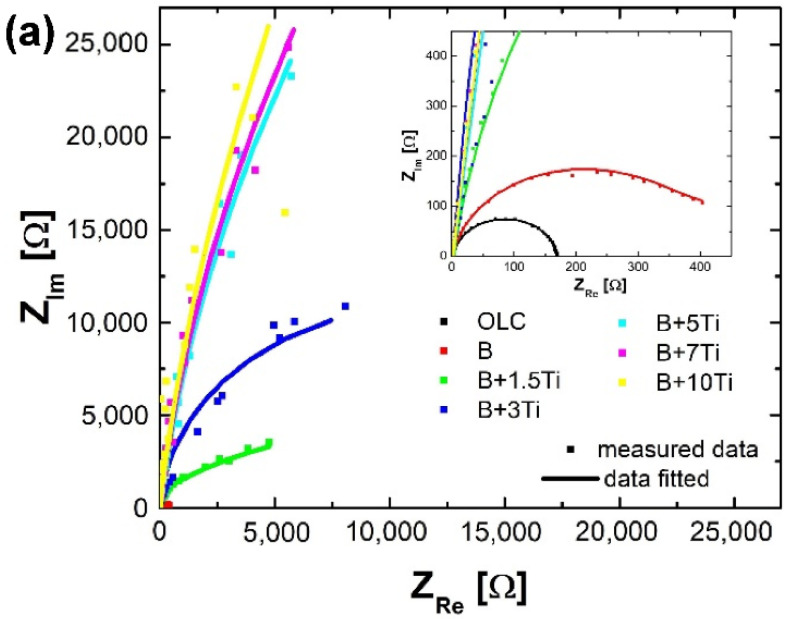

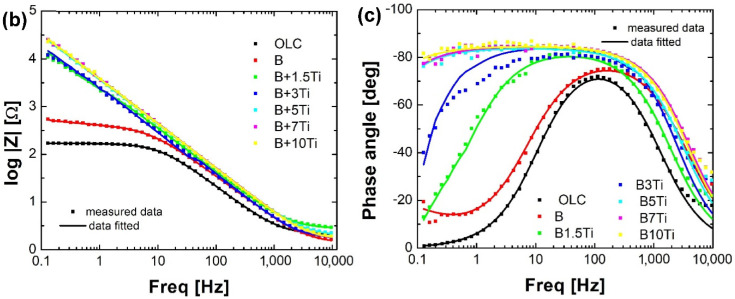
Nyquist (**a**) and Bode plots (**b**,**c**) of mild steel and NiCr(Ti) coatings on mild steel in H_2_SO_4_ 0.5 M (pH = 0.3) + 2 ppm F^−^ at 25 °C.

The EIS measurements were conducted at open circuit value at room temperature. In Figure 7 are presented the corresponding Nyquist and Bode plots of mild steel and NiCr(Ti) coatings on a mild steel substrate. All Nyquist plots (see Figure 7a) are presented to show a single capacitive loop with a low diameter for mild steel and base material (NiCr protective coating). A higher spectra diameter for coated samples with 5%, 7% and 10% Ti addition can be observed, which is in accordance with the obtained values of corrosion parameters from the potentiodynamic polarization curves.

As the titanium content increases, the materials are still very stable in H_2_SO_4_ 0.5M (pH = 0.3) + 2 ppm F^−^ at 25 °C and the radius of the semicircle in the Nyquist plot exhibits a substantial increment, which means an improvement in the polarization resistance (*R_p_*) or, alternatively, an increment in the corrosion resistance.

In the Bode-|Z| diagrams (see Figure 7b), a strong shift of the impedance modulus to higher values is noted as the titanium content increases, which clearly indicates an increase in the corrosion resistance because of the development of the passive film on the surface of the analyzed samples. The |Z| values in the curves corresponding to +5% Ti, +7% Ti and +10% Ti are very close and slightly higher than those obtained at 1.5% and 3% Ti because of the increased thickness of the passive film with the increment of the titanium content. The slopes of the graphics follow the value −1 for all the samples, which indicates the capacitive performance of the passive layer that is built up.

In the Bode phase plots (see Figure 7c), a characteristic pattern of the early nucleation of a passive film on the surface of the sample can be seen. As the titanium content increases by more than 3%, the film formed thickens and has capacitive behavior demonstrated by a phase angle approaching 90° over a large band of frequencies, a process which is linked to a decrease in capacitance.

After analyzing the impedance spectra profiles, the collected experimental results will be fitted to an equivalent electrical circuit. An equivalent circuit consists of a group of elements (resistors, capacitances, Warburg elements, inductors and other impedance distribution components) that provide a similar response to corrosion in the analyzed frequency interval.

When the laboratory impedance data are analyzed, they are compared with the performance of an equivalent electrical circuit and the measurement of the values of the individual electrical parameters is performed. When corrosion processes are involved, these values serve to provide information on the corrosion resistance of the material as well as on the corrosion process mechanism. When considering the application of equivalent electrical circuits for the analysis of EIS data, it is important to realize that there is often a great variety of circuit arrangements that can very accurately replicate the identical behavior as that found experimentally in a real process.

**Figure 8:**
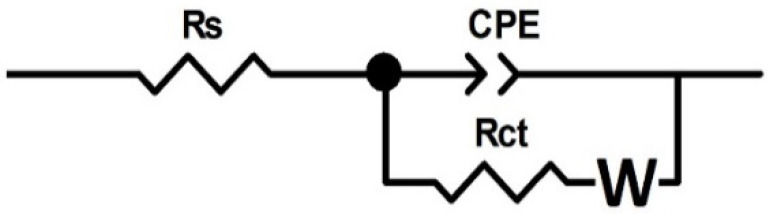
Equivalent circuit for spectra fitting.

In Figure 8, the equivalent circuit used to fit the impedance spectrum of samples measured at OCP is presented. The circuit is composed of the following: R_s_ as the 0.5 M H_2_SO_4_ + 2 ppm F^−^ solution resistance, R_ct_ as the charge-transfer resistance at the interface electrolyte/coating, W as Warburg resistance and CPE as the constant phase element. This constant phase element has been selected in place of an ideal capacitance in order to consider the inhomogeneities of the passive layer [32].

Table 4 shows the calculated values of the circuit elements for modeling the coated samples. The higher R_ct_ suggest an enhanced corrosion resistance.

The impedance of a CPE is given by the following [33]:

(4) $$Q=Z_{CPE\;}\left(\omega \right)={\left[C{\left(j\omega \right)}^{n}\right]}^{-1}\;$$

A parameter obtained by analyzing the process is the ideality factor “*n*”, which means that the performance of the real process is more similar to the ideal, since the value of *n* is nearer to the unit and, consequently, the surface area is more uniform.

The finite Warburg diffusional element has the impedance *Z_w_* given by the following [34]:

(5) $$Z_{W}=R_{D}\frac{tanh\sqrt{jw\tau_{D}}}{\sqrt{jw\tau_{D}}}$$

where: *w* is the frequency (Hz), *j* is the imaginary unit, *τ_D_* is a time constant associated with the mass transfer and *R_D_* is the resistance of the finite Warburg diffusional element. The existence of the Warburg resistance is related to the corrosion process controlled by the diffusion circulation.

## 4. Conclusions

A NiCr coating with different Ti additions was successfully deposited on mild steel plates by laser cladding technology in order to be used as potential materials for bipolar plates in polymer electrolyte membrane fuel cells. Optical microscopy, SEM, EDS, XRD, OCP, LP and EIS were performed in order to compare the microstructure and behavior of the coatings in a solution consisting of 0.5 M H_2_SO_4_ + 2 ppm F^−^ at room temperature. The results lead to the following conclusions:

- All the analyzed coatings show a dendritic microstructure; the size of dendrites increased with the Ti addition while the volume fraction of the NiCr-ɣ phase diminished;
- The dendritic region is rich in Ni, Cr and Mo with traces of Fe and Ti, while the inter-dendritic area is rich in Ni, Cr, Mo and Nb, also with traces of Ti and Fe;
- In the coatings manufactured with Ti addition, the presence of Ti within the coatings was confirmed by XRD; titanium is dissolved into the FCC Ni-Cr phase and the new Ni-Cr-Ti phase is formed;
- For all the coatings on mild steel, the OCP changes to more positive values in time, which is typically attributed to the growth and stabilization of a passive film on the surface of the samples;
- The decrease in the anodic current densities with the increase in titanium addition in the NiCr(Ti) coatings indicates an increase in corrosion resistance in the aggressive environment used in experimental tests; the values of the cathodic current densities decrease proportionally with the amount of titanium added in coatings, which means that in the acidic environment used to carry out the tests, titanium acts as an inhibitor of the cathodic process, thus limiting the hydrogen evolution reaction;
- The protective efficiency increases with the increase in Ti content due to the formation of a stable oxide film on the surface that enhances the corrosion resistance in an aggressive acidic medium;
- The corrosion rates decrease with the increase in Ti content due to the formation of the thin oxide film developed on the coating’s surface;
- As the titanium content increases more than 3%, the formed film thickens and has capacitive behavior, a process which is linked to a decrease in capacitance;
- The coatings with 5% Ti, 7% Ti and 10%Ti addition comply with the conditions of the US DOE regarding corrosion performance to be used as materials for the manufacture of the bipolar plates.

## Figures and Tables

**Table 1:** Parameters for laser cladding deposition of samples.

Samples	Powder Preheating [°C]	Type of Laser	Power [W]	Focal Distance [mm]	Stand-Off Distance [mm]	Deposition Speed [cm/min]	Gas Flow [L/min]
B B + 1.5% Ti B + 3% Ti B + 5% Ti B + 7%Ti B + 10% Ti	70	continuous	859	200	11.7	40	15.1

**Table 2:** Polarization parameters for samples tested in H_2_SO_4_ 0.5 M (pH = 0.3) + 2 ppm F^−^ solution.

Samples	*i_corr_* [μA·cm^−2^]	*E_corr_* [mV/SCE]	−*b_c_* [mV·dec^−1^]	*b_a_* [mV·dec^−1^]	*R_p_* [kΩ·cm^2^]
OLC	46.31	−575	75.2	50	0.28
OLC-B (B)	9.84	−395	72.1	451	2.74
B + 1.5% Ti	2.41	−325	29.1	422	4.91
B + 3% Ti	1.72	−292	27.7	410	6.55
B + 5% Ti	0.189	−239	25.4	394	54.82
B + 7% Ti	0.085	−216	23.2	385	111.77
B + 10% Ti	0.071	−206	21.3	373	123.22

**Table 3:** Protection efficiency (*PE*) and corrosion rates (*CR*) for mild steel and NiCr(Ti) coatings on mild steel in H_2_SO_4_ 0.5 M (pH = 0.3) + 2 ppm F^−^ at 25 °C.

Samples	*PE* [%]	*CR* [mm/yr]
OLC	-	0.5423
OLC-B (B)	78.7	0.0980
B + 1.5%Ti	94.8	0.0235
B + 3%Ti	96.3	0.0163
B + 5%Ti	99.5	0.0017
B + 7%Ti	99.8	0.0007
B + 10%Ti	99.8	0.0006

**Table 4:** Calculated values of the circuit elements for modeling of metallic samples in test solution.

Samples	R_s_ [Ω·cm^2^]	*Q* [S·cm^−2^·s^n^]	*n*	R_ct_ [Ω·cm^2^]	W [S·cm^−2^·s^0.5^]	Chi^2^
OLC	2.073	12.11 × 10^−5^	0.9251	168	7.958 × 10^−9^	1.84 × 10^−3^
OLC-B (B)	1.364	8.61 × 10^−5^	0.9005	385	5.946 × 10^−3^	1.26 × 10^−3^
B + 1.5% Ti	2.784	6.01 × 10^−5^	0.9251	4005	1.789 × 10^−3^	2.90 × 10^−3^
B + 3% Ti	1.807	5.13 × 10^−5^	0.9634	2.293 × 10^4^	9.696 × 10^−5^	4.22 × 10^−2^
B + 5% Ti	1.661	4.61 × 10^−5^	0.9404	2.694 × 10^4^	2.694 × 10^−5^	5.63 × 10^−3^
B + 7% Ti	1.589	4.54 × 10^−5^	0.9397	1.861 × 10^5^	4.301 × 10^−5^	2.51 × 10^−3^
B + 10% Ti	1.972	4.87 × 10^−5^	0.9329	1.909 × 10^5^	1.587 × 10^−4^	2.25 × 10^−3^

## Data Availability

Not applicable.
